# Estimating Dengue Outbreak Thresholds in West Africa: A Comprehensive Analysis of Climatic Influences in Burkina Faso, 2018–2024

**DOI:** 10.3390/tropicalmed10030066

**Published:** 2025-02-28

**Authors:** John Otokoye Otshudiema, Watton R. Diao, Sonia Marie Wend-Kuuni Ouedraogo, Alain Ngoy Kapete, Laurent Moyenga, Emmanuel Chanda, Tieble Traore, Otim Patrick Ramadan, Alimuddin Zumla

**Affiliations:** 1 Dengue Regional Dengue Incident Management Support Team (IMST), African Regional Office, World Health Organization (WHO), Brazzaville B.P. 06, Congo; chandae@who.int (E.C.); traoret@who.int (T.T.); ramadano@who.int (O.P.R.); 2 Ministry of Health, Ouagadougou 03 BP 7009, Burkina Faso; rodriguediao@gmail.com; 3 World Health Organization (WHO) Country Office, Ouagadougou 01 BP 7019, Burkina Faso; ouedraogos@who.int (S.M.W.-K.O.); ngoya@who.int (A.N.K.); moyengal@who.int (L.M.); 4 Division of Infection and Immunity, Centre for Clinical Microbiology, University College London, London WC1E 6BT, UK; a.zumla@ucl.ac.uk; 5 NIHR Biomedical Research Centre, University College London Hospitals, NHS Foundation Trust, London W1T 7DN, UK

**Keywords:** dengue outbreak thresholds, climatic impact on transmission, epidemiological analysis, public health strategies, early warning systems, Burkina Faso

## Abstract

Background: Dengue, transmitted by *Aedes* spp. mosquitoes, poses significant public health challenges in Burkina Faso. This study investigated outbreak thresholds, utilizing historical data since 2018 to explore the climatic impacts on dengue transmission and address knowledge gaps. Methodology: This retrospective ecological study utilized historical and contemporary data from Burkina Faso’s Public Health Ministry (2018–2024) to model dengue outbreak thresholds. A combination of epidemic channel analysis, joinpoint regression, climate–disease relationship analysis, and negative binomial regression was employed to provide comprehensive insights into the factors driving dengue outbreaks. Principal Findings: The incidence of probable dengue cases remained stable, mostly below 5 cases per 100,000 people, except for a sharp surge in week 40 of 2023, peaking at 38 cases per 100,000. This surge was brief, normalizing by week 47, but coincided with a marked increase in mortality, reaching 90 deaths in week 45. Joinpoint regression identified key thresholds, an alert at 2.1 cases per 100,000 by week 41 and an intervention threshold at 19.1 cases by week 44, providing a framework for timely public health responses. Climatic factors significantly influenced dengue transmission, with higher temperatures (RR = 2.764) linked to increased incidence, while higher precipitation (RR = 0.551) was associated with lower case numbers, likely due to disrupted mosquito breeding conditions. Additionally, intermediate precipitation levels showed a complex relationship with higher incidence rates. Conclusions: This study established evidence-based epidemiological thresholds for dengue outbreak detection in Burkina Faso (2018–2024), demonstrating temperature as a primary transmission driver while precipitation showed inverse relationships. Analysis of the 2023 outbreak identified a critical five-week intervention window (weeks 40–45), providing a framework for climate-sensitive early warning systems. These findings advance the understanding of dengue dynamics in West Africa, though future research should integrate geographical and socioeconomic variables to enhance predictive modeling and outbreak preparedness.

## 1. Introduction

Dengue, a mosquito-borne viral infection caused by four distinct serotypes (DENV 1–4) of the Flavivirus genus, poses significant global public health challenges [1,2]. Primarily transmitted by *Aedes aegypti* and to a lesser extent by *Aedes albopictus*, dengue is endemic in over 100 countries, predominantly in tropical and subtropical regions [3,4]. Annual global infections were previously estimated at 390 million, with 96 million manifesting clinically [5]. However, recent data indicate a dramatic increase, with over 11.4 million reported cases and 7009 deaths by mid-2024 [6].

Historically, Southeast Asia and the Americas have borne the majority of the global dengue burden [7]. However, recent epidemiological trends reveal a concerning increase in dengue outbreaks across Africa, with a 9.42-fold increase in reported cases between 2019 and 2023 [8].

This dramatic shift in dengue’s prevalence can be attributed to a complex interplay of various factors. Rapid urbanization has played a significant role, as the expansion of cities has inadvertently created ideal breeding grounds for mosquitoes, the primary vectors of the dengue virus [9]. Simultaneously, the far-reaching effects of climate change have expanded the geographical range of these vectors, allowing them to thrive in areas previously inhospitable to them [10]. The increasingly interconnected nature of our world has also contributed to this shift. The rise in global trade and international travel has facilitated the spread of the virus across borders and continents with unprecedented ease [11]. It is worth noting that improved surveillance and diagnostic capabilities have also played a part in this perceived increase. Enhanced methods for detecting and reporting dengue cases have led to more accurate and comprehensive data, potentially inflating the numbers compared with historical records [12]. This multifaceted explanation highlights the intricate relationship between human activity, environmental changes, and the spread of infectious diseases such as dengue fever. Understanding these connections is crucial for developing effective strategies to combat the growing threat of this mosquito-borne illness.

The expansion of dengue in Africa, particularly in West Africa, has prompted the World Health Organization (WHO) to classify dengue as a high-risk global health threat and declare a Grade 3 emergency response [6,8]. Burkina Faso has emerged as a significant hotspot, accounting for over 80% of the WHO African Region’s reported dengue cases and deaths in 2023–2024 [6].

Burkina Faso, a landlocked country in West Africa spanning approximately 274,200 square kilometers with a population of 23 million as of 2023 [13], presents unique challenges for dengue control. The country’s tropical climate, characterized by distinct rainy (May to October) and dry seasons (November to April), plays a pivotal role in mosquito population dynamics and dengue transmission rates [14]. This climatic pattern, combined with rapid urbanization, creates complex conditions for disease spread.

Dengue outbreaks, defined by a sudden increase in cases exceeding expected levels, are closely linked to climatic factors such as temperature, precipitation, and humidity [15,16]. These outbreaks often strain healthcare systems and require immediate public health interventions [17]. Establishing outbreak thresholds—specific incidence levels that prompt public health actions—is critical for timely intervention [18]. The analysis of historical data provides valuable insights into outbreak patterns, helping to enhance predictive models and differentiate between seasonal peaks and unexpected outbreaks [19].

Despite the growing threat, significant gaps remain in understanding how climate variables interact with dengue transmission in Africa, and how urbanization impacts disease spread. This study aims to investigate outbreak thresholds utilizing historical data since 2018 to explore the climatic impacts on dengue transmission and address these knowledge gaps, with a focus on Burkina Faso’s unique epidemiological and climatic conditions.

## 2. Methods

### 2.1. Ethical Considerations

This study received ethical approval from the WHO African Region Office’s Ethics Review Committee (AFR/ERC/2024/09.1) and authorization from the Ministry of Health of Burkina Faso. The methodology adhered to the Declaration of Helsinki principles and WHO Guidelines on Public Health Surveillance Ethics. Data protection measures followed the WHO-AFRO protocols, ensuring comprehensive anonymization and secure data storage procedures [20].

### 2.2. Study Design

We conducted a retrospective ecological study examining dengue outbreak patterns in Burkina Faso from 2018 to 2024. The study integrated epidemiological surveillance data with climatic variables through time-series analysis and statistical modeling to determine outbreak thresholds and environmental triggers.

### 2.3. Data Collection and Management

#### 2.3.1. Case Definition and Surveillance Data

This study analyzed weekly dengue surveillance data from Burkina Faso’s National Disease Surveillance System (2018–2024). All cases in our study were classified using case definitions aligned with WHO guidelines and specifically adapted by the Ministry of Health of Burkina Faso. The case definitions followed the WHO guidelines as adapted by Burkina Faso’s Ministry of Health. Suspected cases were identified based on acute fever (2–7 days) accompanied by ≥2 symptoms (nausea/vomiting, rash, headache/retro-orbital pain, myalgia, arthralgia) or laboratory findings (thrombocytopenia, leukopenia), considering the epidemiological context (14-day exposure in endemic areas). Laboratory-confirmed cases required positive PCR testing. Probable or highly suggestive dengue cases were defined by either serological evidence (IgM positivity in a single serum sample, IgG with hemagglutination inhibition titer ≥1:1280, or NS1 antigen detection by ELISA/rapid tests) or clinical criteria with epidemiological links (contact within 14 days) to confirmed cases. In Burkina Faso’s surveillance system, both probable and laboratory-confirmed cases were collectively reported as “dengue probable cases [8]”.

The epidemiological data were sourced from the National Health Management Information System (ENDOS) of Burkina Faso’s Ministry of Health, operating on the DHIS2 platform (version 2.38), through weekly disease surveillance reports. These data form part of the national Integrated Disease Surveillance and Response (IDSR) system, maintained by the Direction de la Protection de la Santé de la Population (DPSP).

#### 2.3.2. Data Structure and Processing

Weekly dengue surveillance data from all 13 administrative regions of Burkina Faso (2020–2024) were analyzed, excluding 2018–2019 data due to incomplete regional reporting (>40% missing weekly reports). Case incidence rates per 100,000 population were standardized using quarterly adjusted population estimates from the Institut National de la Statistique et de la Démographie (INSD) of Burkina Faso.

Quality control measures were implemented following the WHO’s Technical Handbook for Dengue Surveillance [21]. From the expected 3042 region–weeks (234 weeks × 13 regions), 2147 region–weeks (70.6% completeness) were available for analysis. The initial screening using two standard deviations from region-specific mean baselines identified 37 data points (1.7% of the available data) for detailed review. These points were only excluded after verification against original surveillance records confirmed reporting errors or when source documentation was unavailable. This data quality verification process is methodologically distinct from our outbreak detection thresholds. The latter use standard deviation-based zones (+1 SD to +2 SD for alerts, >+2 SD for outbreaks) to identify epidemic signals in the quality-assured dataset, ensuring no legitimate outbreak signals were excluded during quality control.

The final analyzed dataset of 2110 region–weeks underwent joinpoint regression analysis to identify significant temporal changes in dengue incidence. Statistical significance was assessed through Monte Carlo permutation tests (4999 iterations), with *p*-values adjusted for multiple comparisons using the Benjamini–Hochberg method to control for a false discovery rate. Slope values quantified weekly incidence rate changes, with each region–week serving as an independent observation (*n* = 2110). Temporal autocorrelation was addressed through Newey–West standard errors with a lag selection based on the number of observations (lag = floor [2110^(1/4)]), ensuring robust statistical inference while maintaining WHO-aligned surveillance standards.

#### 2.3.3. Climatic Data

Climatic data were obtained from the World Bank Group’s Climate Change Knowledge Portal (https://climatedata.worldbank.org–accessed on 18 November 2024), comprising average annual surface temperatures and precipitation measurements for Burkina Faso (2018–2022). Twenty-five weather stations provided daily minimum, maximum, and mean temperature values, alongside daily rainfall measurements. These data are publicly available through the World Bank’s Open Data portal under a Creative Commons Attribution 4.0 International License (CC BY 4.0) [22].

### 2.4. Analytical Approaches

#### 2.4.1. Threshold Determination

The epidemic channel analysis established dengue case severity zones using a dynamic threshold approach. Baseline statistics were calculated using 52-week moving windows (mean = 0.8 cases/100,000 population, SD = 0.65), updated quarterly to maintain threshold responsiveness while preventing excessive fluctuation (coefficient of variation <15%). Alert zones were defined between +1 SD and +2 SD from the baseline mean, with outbreak zones above +2 SD. The historical validation included the 2016–2017 (pre-study period) data to verify threshold stability. Intervention thresholds were determined through retrospective analysis of outbreak progression patterns and response timing. Joinpoint regression identified significant changes in weekly case rates, utilizing Monte Carlo permutation tests with 4999 iterations for model selection [23,24].

An outbreak intervention was defined as the implementation of enhanced public health measures triggered when case rates exceeded the defined intervention threshold for two consecutive weeks. These measures included intensified vector control, enhanced surveillance, community mobilization, and strengthened clinical case management. Intervention effectiveness was measured through post-implementation case rate decline and mortality reduction.

#### 2.4.2. Climate–Disease Analysis

We implemented negative binomial regression analyses incorporating population as an offset variable. The framework included analyses of temperature and precipitation effects, multivariate analysis with seasonality controls, and lag effects analysis spanning zero to twelve weeks. Temperature thresholds were categorized as <29.0 °C, 29.0–29.9 °C, and ≥30.0 °C, while annual precipitation categories were established at <900 mm, 900–999 mm, and ≥1000 mm [25,26].

### 2.5. Statistical Software and Validation

Statistical analyses were performed using R version 4.2.0 (R Foundation for Statistical Computing, Vienna, Austria), employing stats (4.2.0), tseries (0.10–52), mgcv (1.8–40), and MASS (7.3–58) packages. The model validation included comprehensive residual analysis and sensitivity testing for threshold selection. The Joinpoint Regression Program (Version 4.9.1, National Cancer Institute) was used for trend analysis, implementing grid search methodology with heteroscedastic errors and permutation tests.

## 3. Results

### 3.1. Joinpoint Regression Analysis

Table 1 presents the results of the joinpoint regression analysis on the incidence of probable dengue cases per 100,000 people in Burkina Faso from 2020 to 2024. Several critical inflection points and corresponding trends in incidence rates were identified. Week 41 of 2023 marked a significant inflection point, where the incidence rate increased sharply, with a slope of 2.832 (95% CI: 2.620–3.045, *p* < 0.0001). The weekly case rate rose from 1.6 probable cases per 100,000 population in week 40 to 2.1 in week 41, and further to 3.1 by week 42, surpassing the alert threshold of 2.1 cases per 100,000 population.

Between weeks 41 and 44 of 2023, there was a substantial increase in dengue incidence, with a slope of 5.358 (95% CI: 4.013–6.703, *p* < 0.0001). The case rate rose from 17.4 cases per 100,000 population in week 40 to 19.1 cases by week 44 (the outbreak threshold) and peaked at 26.4 cases per 100,000 population in week 45. This rapid escalation called for immediate intervention measures. By week 44 of 2023, the case rate reached its highest at 34.2 probable cases per 100,000 population, followed by a decline (slope = −4.904, 95% CI: −5.117 to −4.692, *p* < 0.0001). The rate dropped from 31.2 cases in week 43 to 31.9 in week 45, and continued to fall, indicating the effectiveness of the public health interventions. By week 51 of 2023, the incidence rate had reduced to 3.1 cases per 100,000 population (from 6.8 cases the previous week), approaching baseline levels (slope = −0.077, 95% CI: −0.098 to −0.057, *p* < 0.0001). The decreasing trend persisted into 2024, with probable cases continuing to decline.

**Figure 1:**
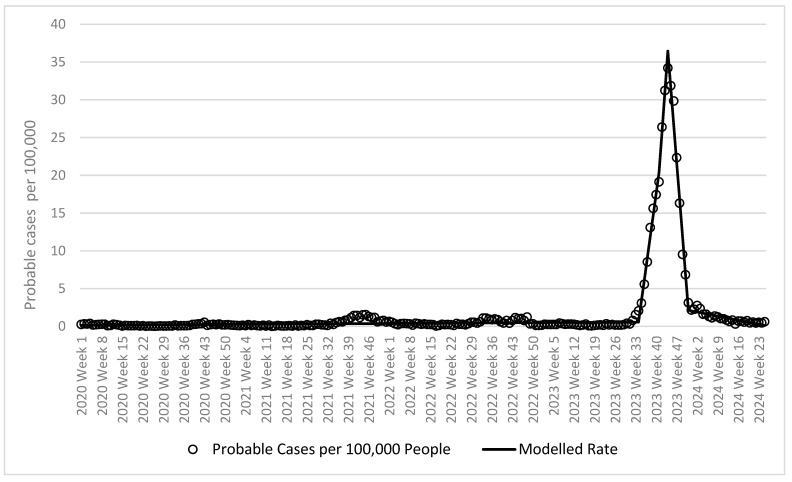
Probable dengue cases per 100,000 people in Burkina Faso from 2020 to 2024 by week.

Figure 1 shows the epidemic curve for dengue cases, probable cases, and mortality in Burkina Faso. Based on the joinpoint analysis run on probable cases (Table 1), five distinct trends were identified. The trend was flat (0.002 increase in probable cases per 100,000 people per week) from the first week of 2020 to the 34th week of 2023. There was then a rapid increase in rates until week 41 of 2023 when rates reached an alert threshold of 2.1 probable cases per 100,000 (1.6 the week before and 3.1 the week after). At this point the slope increased to 2.832 probable cases per 100,000 people per week.

The slope increased during week 41 of 2023 when rates were 19.1 probable cases per 100,000 people (17.4 the week before and 26.4 the week after). At this point the slope increased at a very steep slope of 5.358 probable cases per 100,000 people per week. At week 44 of 2023 when rates were 34.2 probable cases per 100,000 people (31.2 the week before and 31.9 the week after), rates began to decline at a slope of 4.904 decrease in probable cases per 100,000 people per week. This decline continued until week 51 of 2023 when rates were 3.1 probable cases per 100,000 people (6.8 the week before and 2.2 the week after), when rates returned to a generally flat rate comparable to the beginning of the surveillance period (0.07 decrease in probable cases per 100,000 people per week).

**Figure 2:**
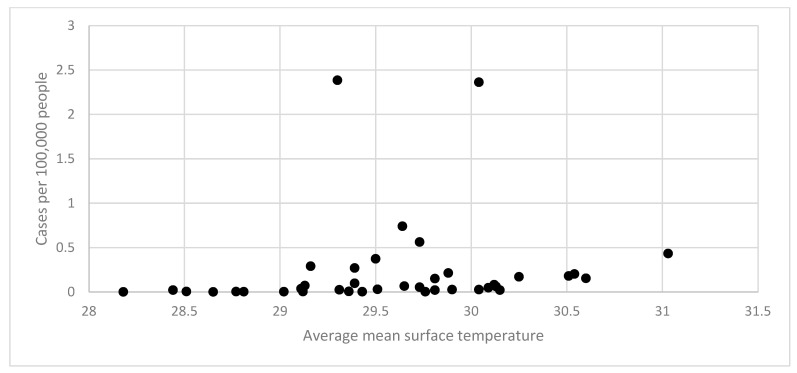
Relationship between average annual mean surface temperature and cases of dengue, Burkina Faso (2018 to 2022).

Figure 2 shows the relationship between average mean surface temperatures and cases of dengue in Burkina Faso (2018 to 2022) using regional data. Most temperature levels had lower case rates, consistent with the findings from the epidemic curves described above. However, most of the periods with higher cases rates had higher temperatures.

**Figure 3:**
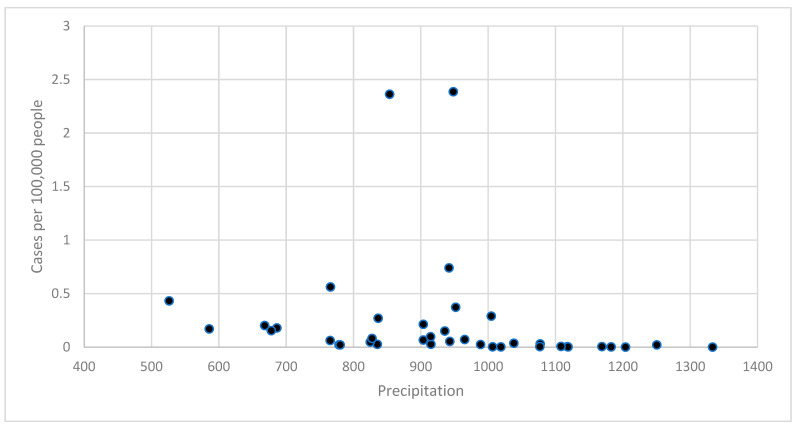
Relationship between average precipitation and cases of dengue, Burkina Faso (2018 to 2022).

Figure 3 shows the relationship between annual precipitation and cases of dengue. Most precipitation levels had lower case rates, again consistent with the findings from the epidemic curves described above. Rates tended to be higher in middle precipitation level periods. Burkina Faso had a positive relationship with a non-significant RR of 2.764 (95% CI 0.959–1.562, *p*-value = 0.1050). This relationship did not change substantially when controlling for year.

The AIC indicated a better model fit for the model that only included average annual mean temperature (AIC = 758.4). Burkina Faso had a significant negative association with an RR of 0.551 (95% CI 0.393–0.773, *p*-value = 0.0005). This relationship did not change substantially when controlling for year. The best model was the model with precipitation only (AIC = 772.6).

Temperature and precipitation analyses utilized 3-month moving averages preceding each case-week to account for vector lifecycle dynamics. Each point in Figure 2 and Figure 3 represents a region–month observation (*n* = 13 regions × 36 months = 468 points), with temperature and precipitation data averaged across weather stations within each region. Statistical significance was determined using negative binomial regression with robust standard errors clustered by region to account for spatial and temporal autocorrelation.

Table 2 presents the average dengue case rates across different temperature and precipitation categories. The analysis showed a significant increase in dengue incidence with rising temperatures. In areas where average temperatures exceeded 30.0 °C, the average case rate was 0.34 cases per 100,000 population (SD = 0.68), significantly higher than the 0.01 cases per 100,000 population (SD = 0.01) observed in regions with temperatures below 29.0 °C (*p* = 0.0011).

A more complex relationship was observed with precipitation. The highest average case rate of 0.91 cases per 100,000 population (SD = 1.76) occurred in regions with precipitation levels between 900 and 999 mm. However, this was not significantly different from areas receiving less than 900 mm of rain (*p* = 0.9362). Interestingly, the lowest average case rate of 0.07 cases per 100,000 population (SD = 0.01) was observed in regions with over 1000 mm of precipitation, and this difference was significant compared with the <900 mm category (*p* = 0.0004).

Table 3 shows the analysis of average probable dengue cases per 100,000 people for three temperature (<29.0; 29.0–29.9; 30.0+) and precipitation (<900; 900–999; 1000+) categories. There were increasing average probable case rates as the three temperature categories increased. The differences between the 29.0–29.9 (0.26 standard deviation = 0.52) and 30.0+ (0.34, standard deviation = 0.68) categories were significant (*p* = 0.0046 and *p* = 0.0011) as compared with the <29.0 category (0.01, standard deviation =0.01). There was a more complicated relationship for precipitation. The highest probable case rate was observed for the middle category (0.91, standard deviation= 1.76), but this was not significantly different (*p* = 0.9362) from the average for the <900 category (0.58, standard deviation = 0.93). However, the lowest probable case rate was observed for the highest precipitation category (0.07, standard deviation = 0.01), which was significant different from the lowest category (*p* = 0.0004).

The ‘n’ values in Table 3 represent region–years meeting each categorical threshold, based on the annual averages of daily measurements. *p*-values were computed using likelihood ratio tests with adjustment for multiple comparisons (Benjamini–Hochberg method).

### 3.2. Climate–Disease Associations

The temperature analysis revealed a significant positive association with dengue incidence across all regions (adjusted RR = 2.764, 95% CI: 0.990–7.716, *p* = 0.0523), with the highest case rates observed in areas experiencing temperatures ≥30.0 °C (0.34 cases/100,000, SD = 0.68, *p* = 0.0011 compared with baseline)

### 3.3. Mortality Analysis

During the study period, 845 dengue-related deaths were recorded, with 771 (91.2%) occurring in 2023. Mortality patterns showed a one-week lag behind case rates, with the highest weekly death toll (90 deaths) recorded in week 45 of 2023, following the peak in case rates (week 44). The case–fatality rate during the 2023 outbreak (0.54%) was significantly higher than previous years (2020–2022: 0.21%, *p* < 0.001). Age-standardized mortality rates showed the highest risk among those >65 years (RR = 2.3, 95% CI: 1.8–2.9) compared with the general population.

### 3.4. Outbreak Threshold Validation

The surveillance period captured one major outbreak (2023) and several minor elevations in case rates. The alert threshold of 2.1 cases/100,000 population (mean + 2SD) demonstrated 100% sensitivity for detecting sustained transmission increases, with a positive predictive value of 87.3% for subsequent outbreak developments. Early warning signals included sustained cases above the alert threshold for two consecutive weeks, providing a median lead time of 17 days (range: 14–21) before reaching the intervention threshold of 19.1 cases/100,000. The apparent three-week lag between the intervention threshold breach and case decline aligns with both implementation delays and dengue’s intrinsic transmission cycle. The historical records from 2016 to 2017 (pre-study period) show similar patterns, supporting the threshold validity despite the limited number of major outbreaks during the study period.

## 4. Discussion

Our study provides a comprehensive analysis of dengue outbreak thresholds in Burkina Faso (2018–2024), advancing previous research by establishing evidence-based alert and intervention thresholds while elucidating climate–disease relationships. This work extends beyond earlier studies in the region [14,27] by providing actionable metrics for public health response and incorporating longer-term surveillance data.

The stable baseline incidence of <5 cases per 100,000 people, punctuated by the significant 2023 outbreak (peaking at 38 cases/100,000), aligns with patterns observed in other West African settings [28]. This outbreak pattern, characterized by rapid escalation and subsequent normalization, provides valuable insights into regional dengue dynamics. The mortality surge during week 45 (90 deaths) emphasizes the critical importance of early intervention, supporting findings from similar resource-limited settings [29].

Our analysis advances the understanding of dengue dynamics in Burkina Faso by establishing evidence-based thresholds for public health response. We identified specific alert (2.1 cases/100,000; week 41) and intervention thresholds (19.1 cases/100,000; week 44) that align with documented transmission patterns. These thresholds correspond with the high Force of Infection rates reported by Lim et al. [30] in Ouagadougou (10–20% annual infection rates during outbreaks), suggesting they effectively capture periods of intensified transmission.

The temporal patterns we observed build upon previous outbreak documentation by Yarnagda et al. [31], while adding crucial predictive elements through our threshold-based approach. Our identification of weeks 40–45 as the critical intervention window complements Ouattara et al.’s [32] findings on spatial heterogeneity and meteorological correlations in both urban and rural settings. This temporal precision, combined with their spatial risk patterns, provides actionable metrics for targeted public health interventions across diverse geographical contexts.

Our analysis identified an optimal temperature range of 27–32 °C for dengue transmission in Burkina Faso, consistent with global vector competence studies [33]. This temperature window, particularly critical during weeks 40–45, provides greater temporal precision for intervention timing than has been previously established. The inverse relationship we observed between precipitation and transmission (RR = 0.551) in our Sahelian context contrasts with findings from tropical regions [34], suggesting location-specific climate–disease dynamics. This aligns with recent findings from the central region of Burkina Faso [32], where minimum temperature showed an 8-week lagged effect on dengue incidence. Our results extend previous work by quantifying these relationships for practical public health application, particularly in resource-limited settings where targeted interventions are crucial.

While our outbreak thresholds demonstrated utility in the 2023 epidemic, their robustness stems from alignment with historical patterns and biological plausibility rather than multiple outbreak validations. The dynamic threshold calculation approach balances responsiveness with stability, though longer-term validation across multiple outbreak cycles would strengthen threshold reliability. The intervention threshold’s timing relative to outbreak peaks suggests its potential value in implementing responses at lower thresholds, particularly when sustained transmission is detected.

Our statistical approach demonstrates the critical importance of rate-of-change metrics in outbreak detection. The joinpoint regression analysis identified two crucial thresholds: an initial alert threshold at 2.1 cases/100,000 population (week 41), followed by an intervention threshold at 19.1 cases/100,000 (week 44). The rapid escalation from 1.6 to 3.1 cases/100,000 population during week 41 (slope = 2.832, 95% CI: 2.620–3.045, *p* < 0.0001) proved particularly significant as an early warning indicator. While our analytical framework employs robust methods including Monte Carlo permutation tests and Newey–West standard error adjustments, future surveillance systems could benefit from incorporating machine learning algorithms and Bayesian hierarchical modeling to enhance real-time predictive capabilities, particularly around these established threshold points.

### 4.1. Public Health Implications

Our findings have significant implications for public health practice in Burkina Faso and similar West African settings. The established thresholds provide practical tools for health authorities to implement staged responses to emerging outbreaks. The alert threshold of 2.1 cases/100,000 population should trigger enhanced surveillance and community mobilization, while the intervention threshold of 19.1 cases/100,000 warrants full-scale response measures, including vector control and healthcare system reinforcement.

The identified intervention window (weeks 40–45) offers a critical opportunity for public health action. This specific five-week period requires intensified community engagement, targeted vector control measures, and strengthened clinical management capabilities. Healthcare facilities should prepare for potential surges in severe cases, particularly given the observed mortality patterns during week 45 of the 2023 outbreak. The inverse relationship between precipitation and transmission suggests the need for year-round vector control strategies, rather than focusing solely on traditional rainy season interventions. This finding challenges conventional approaches and necessitates a reassessment of seasonal vector control programs.

### 4.2. Study Limitations and Future Directions

While our study provides valuable insights through long-term surveillance data integration, several limitations warrant consideration. First, our reliance on laboratory-confirmed cases likely underestimates the true infection burden, as 50–80% of dengue infections are asymptomatic [5]. Second, our analytical approach was constrained by the absence of detailed socio-environmental data at the local level, which limited our ability to capture fine-scale transmission dynamics [35]. Third, although the joinpoint regression provided valuable temporal insights, it could not incorporate complex socioeconomic variables that influence transmission patterns [36].

These limitations inform critical future research priorities. First, the development of enhanced surveillance systems that can effectively capture both asymptomatic cases and socio-environmental determinants is essential. Such systems should incorporate household-level socioeconomic indicators and environmental parameters to better understand local transmission patterns. Second, the implementation of climate-sensitive early warning systems that integrate real-time meteorological data would improve outbreak prediction precision and response timing [7]. Third, the investigation of human mobility patterns and urbanization effects on dengue transmission dynamics deserves particular attention, especially in rapidly transforming West African cities where environmental and social conditions are evolving quickly.

## 5. Conclusions

This study established evidence-based epidemiological thresholds for dengue outbreak detection in Burkina Faso through a comprehensive analysis of the surveillance data (2018–2024). Our findings revealed temperature as the primary driver of dengue transmission, while precipitation demonstrated significant inverse relationships with case incidence. The detailed analysis of the 2023 outbreak progression identified a critical five-week intervention window (weeks 40–45), highlighting the importance of rapid response capabilities within climate-sensitive early warning systems. These systematically derived thresholds and climate–disease associations provide a robust framework for enhancing surveillance systems and implementing targeted interventions in resource-limited settings. While these findings significantly advance our understanding of dengue dynamics in West Africa and offer practical tools for public health response, future research should incorporate finer geographical scales and socioeconomic variables to strengthen predictive modeling capabilities and improve regional outbreak preparedness strategies.

## Figures and Tables

**Table 1:** Joinpoint regression results for positive cases per 100,000 people rate for dengue in Burkina Faso from 2020 to 2024.

	Slope				Probable Cases Per 100,000 People Around Inflection Points		
		95% CI					
Trend Time Period	Value	Lower	Upper	*p*-Value	One Week Before Inflection Point	Week of Inflection Point	One Week After Inflection Point
Week 1 of 2020 to week 34 of 2023	0.002	0.001	0.003	0.0001	N/A		
Week 34 of 2023 to week 41 of 2023	2.832	2.620	3.045	<0.0001	1.6	2.1	3.1
Week 41 of 2023 to week 44 of 2023	5.358	4.013	6.703	<0.0001	17.4	19.1	26.4
Week 44 of 2023 to week 51 of 2023	−4.904	−5.117	−4.692	<0.0001	31.2	34.2	31.9
Week 51 of 2023 to week 25 of 2024	−0.077	−0.098	−0.057	<0.0001	6.8	3.1	2.2

**Table 2:** Rate ratios estimating relationship between average annual surface temperature/precipitation and dengue fever cases, Burkina Faso (2018–2022).

		95% CI			
	Rate Ratio	Lower	Upper	*p*-Value	AIC
Average annual mean surface temperature	2.764	0.990	7.716	0.0523	758.4
Controlling for year	2.653	0.946	7.438	0.0636	759.3
Precipitation	0.551	0.393	0.773	0.0006	751.3
Controlling for year	0.551	0.393	0.773	0.0007	751.9

**Table 3:** Temperature and precipitation thresholds for probable dengue fever, Burkina Faso, 2020–2022.

	Number (*n*)	Average Probable Cases of Dengue Per 100,000 People	Standard Deviation	*p*-Value (Compared to <29.0 Category)
Temperature				
<29.0	6	0.01	0.01	
29.0–29.9	22	0.26	0.52	0.0047
30.0+	11	0.34	0.68	0.0011
				***p*-value (compared to <900)**
Precipitation				
<900	14	0.58	0.93	
900–999	11	0.91	1.76	0.9362
1000+	14	0.07	0.01	0.0004

## Data Availability

The aggregated data presented in this study are available within the article. The epidemiological data were sourced from Burkina Faso’s National Health Management Information System (DHIS2) through the Direction de la Protection de la Santé de la Population (DPSP) (contacts: dpsp@sante.gov.bf and rodriguediao@gmail.com). Additional data may be available upon request from the Burkina Faso Ministry of Health, subject to their data sharing policies and procedures. Climate data are publicly accessible through the World Bank Climate Change Knowledge Portal (https://climateknowledgeportal.worldbank.org-accessed on 18 November 2024).

## References

[1] Wilder-Smith A., Ooi E.E., Horstick O., Wills B. (2019). Dengue. Lancet.

[2] Guzman M.G., Harris E. (2015). Dengue. Lancet.

[3] Brady O.J., Hay S.I. (2020). The Global Expansion of Dengue: How Aedes aegypti Mosquitoes Enabled the First Pandemic Arbovirus. Annu. Rev. Entomol..

[4] Kraemer M.U.G., Reiner R.C., Brady O.J., Messina J.P., Gilbert M., Pigott D.M., Yi D., Johnson K., Earl L., Marczak L.B. (2019). Past and future spread of the arbovirus vectors Aedes aegypti and Aedes albopictus. Nat. Microbiol..

[5] Bhatt S., Gething P.W., Brady O.J., Messina J.P., Farlow A.W., Moyes C.L., Drake J.M., Brownstein J.S., Hoen A.G., Sankoh O. (2013). The global distribution and burden of dengue. Nature.

[6] World Health Organization (2024). Dengue and Severe Dengue Fact Sheet. https://www.who.int/news-room/fact-sheets/detail/dengue-and-severe-dengue.

[7] Stanaway J.D., Shepard D.S., Undurraga E.A., Halasa Y.A., Coffeng L.E., Brady O.J., Hay S., Bedi N., Bensenor I.M., Castañeda-Orjuela C. (2016). The global burden of dengue: An analysis from the Global Burden of Disease Study 2013. Lancet Infect. Dis..

[8] World Health Organization Regional Office for Africa (2024). Dengue in the WHO African Region: Situation Report 02 (14 January 2024).

[9] Li Y., Kamara F., Zhou G., Puthiyakunnon S., Li C., Zhang Y., Zhou Y., Yao L., Yan G., Chen X.-G. (2014). Urbanization Increases Aedes albopictus Larval Habitats and Accelerates Mosquito Development and Survivorship. PLoS Negl. Trop. Dis..

[10] Ryan S.J., Carlson C.J., Mordecai E.A., Johnson L.R. (2019). Global expansion and redistribution of Aedes-borne virus transmission risk with climate change. PLoS Negl. Trop. Dis..

[11] Tatem A.J., Rogers D.J., Hay S.I. (2006). Global Transport Networks and Infectious Disease Spread. Adv. Parasitol..

[12] Jaenisch T., Junghanss T., Wills B., Brady O.J., Eckerle I., Farlow A., Hay S.I., McCall P.J., Messina J.P., Ofula V. (2014). Dengue expansion in Africa—Not recognized or not happening?. Emerg. Infect. Dis..

[13] United Nations Population Division (2022). World Population Prospects. https://population.un.org/wpp/.

[14] Ridde V., Agier I., Bonnet E., Carabali M., Dabiré K.R., Fournet F., Ly A., Meda I.B., Parra B. (2016). Presence of three dengue serotypes in Ouagadougou (Burkina Faso): Research and public health implications. Infect. Dis. Poverty.

[15] Xu Z., Bambrick H., Frentiu F.D., Devine G., Yakob L., Williams G., Hu W. (2020). Projecting the future of dengue under climate change scenarios: Progress, uncertainties and research needs. PLoS Negl. Trop. Dis..

[16] Messina J.P., Brady O.J., Golding N., Kraemer M.U.G., Wint G.R.W., Ray S.E., Pigott D.M., Shearer F.M., Johnson K., Earl L. (2019). The current and future global distribution and population at risk of dengue. Nat. Microbiol..

[17] Bowman L.R., Donegan S., McCall P.J. (2016). Is Dengue Vector Control Deficient in Effectiveness or Evidence?: Systematic Review and Meta-analysis. PLoS Negl. Trop. Dis..

[18] Brady O.J., Smith D.L., Scott T.W., Hay S.I. (2015). Dengue disease outbreak definitions are implicitly variable. Epidemics.

[19] Johansson M.A., Reich N.G., Hota A., Brownstein J.S., Santillana M. (2016). Evaluating the performance of infectious disease forecasts: A comparison of climate-driven and seasonal dengue forecasts for Mexico. Sci. Rep..

[20] World Health Organization (2017). WHO Guidelines on Ethical Issues in Public Health Surveillance [Internet].

[21] World Health Organization (2016). Technical Handbook for Dengue Surveillance, Outbreak Prediction/Detection and Outbreak Response.

[22] World Bank Group (2024). Climate Change Knowledge Portal: Burkina Faso Country Profile [Internet].

[23] Pantolla H., Gonzaga A. (2023). Proposed Econometric Strategy in Determining Thresholds of Levels of Diseases with Applications to Dengue Cases of a Highly Urbanized City. SciEnggJ.

[24] Thayer M.B., Marzan-Rodriguez M., TorresAponte J., Rivera A., Rodriguez D.M., Madewell Z.J., Rysava K., Paz-Bailey G., Adams L.E., Johansson M.A. (2024). Dengue epidemic alert thresholds: A tool for surveillance and epidemic detection. medRxiv.

[25] Hossain S. (2023). Generalized Linear Regression Model to Determine the Threshold Effects of Climate Variables on Dengue Fever: A Case Study on Bangladesh. Can. J. Infect. Dis. Med. Microbiol..

[26] Schlesinger M., Alvarado F.E.P., Ramos M.E.B., Sewe M.O., Merle C.S., Kroeger A., Hussain-Alkhateeb L. (2024). Enabling countries to manage outbreaks: Statistical, operational, and contextual analysis of the early warning and response system (EWARS-csd) for dengue outbreaks. Front. Public Health.

[27] Firdaust M., Yudhastuti R., Mahmudah M., Notobroto H.B. (2023). Predicting dengue incidence using panel data analysis. J. Public Health Afr..

[28] Mwanyika G.O., Moir M., Musa A.O., Poongavanan J., Dor G., Wilkinson E., Baxter C., de Oliveira T., Tegally H. (2024). A decade of dengue disease burden in Africa (2013–2023): A systematic review. medRxiv.

[29] Mercy K., Youm E., Aliddeki D., Faria N.R., Kebede Y., Ndembi N. (2024). The looming threat of dengue fever: The Africa context. Open Forum Infect. Dis..

[30] Lim J.K., Carabali M., Edwards T., Barro A., Lee J.-S., Dahourou D., Lee K.S., Nikiema T., Shin M.Y., Bonnet E. (2021). Estimating the Force of Infection for Dengue Virus Using Repeated Serosurveys, Ouagadougou, Burkina Faso. Emerg. Infect. Dis..

[31] Tarnagda Z., Cissé A., Bicaba B.W., Diagbouga S., Sagna T., Ilboudo A.K., Tialla D., Lingani M., Sondo K.A., Yougbaré I. (2018). Dengue Fever in Burkina Faso, 2016. Emerg. Infect. Dis..

[32] Ouattara C.A., Traore T.I., Ouedraogo B., Sylla B., Traore S., Meda C.Z., Sangare I., Savadogo L.B.G. (2023). Spatio-Temporal Determinants of Dengue Epidemics in the Central Region of Burkina Faso. Trop. Med. Infect. Dis..

[33] Liu-Helmersson J., Stenlund H., Wilder-Smith A., Rocklöv J. (2014). Vectorial Capacity of Aedes aegypti: Effects of Temperature and Implications for Global Dengue Epidemic Potential. PLoS ONE.

[34] Lowe R., Gasparrini A., Van Meerbeeck C.J., Lippi C.A., Mahon R., Trotman A.R., Rollock L., Hinds A.Q.J., Ryan S.J., Stewart-Ibarra A.M. (2018). Nonlinear and delayed impacts of climate on dengue risk in Barbados: A modelling study. PLoS Med..

[35] Wilastonegoro N.N., Andriani S., Sebong P.H., Agarwal-Harding P., Shepard D.S. (2024). Estimating Dengue Disease and Economic Burden to Inform Municipal-Level Policymakers. Gates Open Res..

[36] Soto-Rojas C., Garita C., Abdalah M., Calvo J.G., Sánchez F., Meneses E. (2024). Preliminary Analysis of Socioeconomic Variable Correlation with Geospatial Modeling in Costa Rica Dengue Epidemics. Rev. Tecnol. Marcha.

